# Differences in the aging of the spine according to physical activity levels in older women

**DOI:** 10.3389/fmed.2025.1730935

**Published:** 2026-01-13

**Authors:** Jessica Brusa, Valerio Giustino, Giuseppe Messina, Ligia Juliana Dominguez, Dorota Kostrzewa-Nowak, Mario Barbagallo, Robert Nowak, Ignazio Leale, Antonino Patti, Antonino Bianco, Giuseppe Battaglia, Angelo Iovane, Elvira Padua

**Affiliations:** 1 Department of Human Sciences and Promotion of the Quality of Life, San Raffaele University, Rome, Italy; 2 Sport and Exercise Sciences Research Unit, Department of Psychology, Educational Science and Human Movement, University of Palermo, Palermo, Italy; 3 Geriatric Unit, Department of Internal Medicine and Geriatrics, University of Palermo, Palermo, Italy; 4 Department of Medicine and Surgery, University Kore, Enna, Italy; 5 Department of Clinical and Molecular Biochemistry, Pomeranian Medical University in Szczecin, Szczecin, Poland; 6 Centre for Human Structural and Functional Research, Institute of Physical Culture Sciences, University of Szczecin, Szczecin, Poland; 7 Department of Pathology, Pomeranian Medical University in Szczecin, Szczecin, Poland

**Keywords:** aging, back, elderly, exercise, physical activity, spinal column, spine, sport

## Abstract

**Introduction:**

The physiological decline with advancing age also affects the aging of the spine. The practice of physical activity (PA) appears to protect against spine degeneration. Hence, the aim of this study was to analyze the morphological differences of the spine in older women, comparing subjects with different levels of PA.

**Methods:**

Participants were divided into the three following groups based on the amount of PA practiced: low active (LA); moderate active (MA); high active (HA). The levels of PA were measured using the International Physical Activity Questionnaire—Short Form (IPAQ–SF). The spine morphology of each participant was assessed through a non-invasive, 3D optoelectronic detection system using the Light Detection and Ranging (LIDAR) technology. Spine parameters in the frontal and sagittal planes were considered for comparisons.

**Results:**

No significant differences in spine parameters in the frontal plane among the 3 groups were found. In the sagittal plane, we found a significant difference on the spine sagittal imbalance parameter (*F*_(2, 40)_ = 6.17; *p* = 0.005), with the highest spine sagittal imbalance in the LA group. Furthermore, in the sagittal plane, we detected a significant difference in the spine inclination parameter (*F*_(2, 40)_ = 5.93; *p* = 0.006), with the highest spine inclination in the LA group.

**Conclusion:**

Our results showed that older women who engage d in lower levels of PA exhibited some altered spinal sagittal parameters compared to peers with moderate and high levels of PA, suggesting that PA may contribute to maintain spinal sagittal alignment and preserve spinal sagittal balance.

## 1 Introduction

Aging is a physiological process which occurs in the human over time and is characterized by progressive and irreversible changes on both the structural and functional domains (1). Among the psychological aspects, the decline in cognitive abilities such as attention and memory represent the most serious issues in old age (2). Whereas, the most common physical conditions are sarcopenia and osteopenia, resulting in reduced functional capacity, compromising daily activities and quality of life (3). The physiological decline with advancing age also affects the aging of the spine which includes the reduction of bone mass and the development of degenerative changes (4). The degeneration of the spine with aging can affect different structures and at various spinal levels including the vertebral body, vertebral endplates, intervertebral discs, facet joints, muscles and ligaments (4, 5). Any alteration to any component, alone or in combination, lead to biomechanical changes which negatively affect range of motion, load carrying capacity, gait and posture (5, 6). Furthermore, this process can lead to several types of lesions and painful symptoms, although the course, speed, and severity of these processes, as well as their consequences, are individual (4).

Considering the prevalence in the population aged over 65, several research groups have shown a growing interest in the topic, by investigating the pathophysiology and biomechanics of the aging spine (4–6). Although this physiological process is dynamic, the extent and speed of these changes are subjective as they can depend on various factors, including the level of physical activity (PA) practice. In fact, the literature is in agreement concerning that regular PA among older women is associated with higher lumbar spine bone mineral density, and that PA programs with higher doses and involving multiple exercises and resistance exercises appear to be more effective (7). For this reason, regular PA appears to protect against spine degeneration, and it is effective to maintain muscle mass and strength, joint mobility, influencing positively body posture (8). Indeed, core muscles have a key role both in the stability and mobility of the spine (4). In contrast, physical inactivity is a risk factor for accelerating the process of musculoskeletal degeneration (9). However, the differences in spine morphology between physically active and sedentary older adults have not yet been extensively investigated.

Radiographic examination is the gold standard for the evaluation of the spine and although in previous decades the evaluation was mainly based on the frontal plane, the importance of the sagittal plane evaluation is now widely recognized (6, 10). Spine assessment includes thorough history, observations, and physical tests which are fundamental to warrant and integrate the radiographic examination (6). However, the use of non-invasive techniques for spine screening can be useful to detect and monitor spine morphology when spine assessment does not justify complementing radiographic examination. In this way, radiation-free examinations are increasingly used to evaluate the spine in order to avoid X-ray exposure (11, 12). Among these alternative methods, the rasterstereography technique, developed by Drerup and Hierholzer (13), represents a valid examination that allows the three-dimensional (3D) reconstruction of the spine by analyzing the back surface (14). In fact, by detecting specific anatomical landmarks of the back surface, the rasterstereography analyzes the 3D back shape and provides the 3D reconstruction of the spine (15). This technique uses the Light Detection and Ranging (LIDAR) technology in which: a sensor sends pulsed laser light (laser emission); a receiver captures the reflected laser light that bounce back from objects (light detection); a processor calculates the time for the pulsed laser light to travel out and back, using the speed of light to measure the distances to the objects; the system creates precise 3D models of objects surface (16). In fact, previous studies assessed the spine morphology through the LIDAR technology (17–20).

Hence, the aim of this study was to analyze the morphological differences of the spine in older women, comparing subjects with different levels of PA. Our hypothesis was that subjects with lower level of PA could present a greater spine imbalance, especially in the sagittal plane.

## 2 Materials and methods

### 2.1 Study design

In this observational cross-sectional study, participants were divided into different groups, based on the amount of PA practiced, and then their spine morphology was assessed to explore any differences.

This study was approved by the Ethics Committee Palermo 1 of the University Hospital “Policlinico di Palermo” (n. 06/2022) and carried out in accordance with the principles of the Declaration of Helsinki.

### 2.2 Participants

Inclusion criteria for participation in the study were as follows: (1) from 65 to 79 years of age; (2) regular participation in any PA or sports for at least 5 years. Exclusion criteria were as follows: (1) presence of any certified spinal deformity; (2) presence of any diagnosed musculoskeletal disease; (3) presence of diagnosed osteoporosis and/or sarcopenia.

A sample of 43 older women were recruited (age: 70.67 ± 3.78 years; weight: 65.22 ± 8.83 kg; height: 1.55 ± 0.08 m).

All participants were informed of the purpose of the study and provided written informed consent to participate.

### 2.3 Procedure

Participants were invited to the Posturology and Biomechanics Laboratory at the University of Palermo and were first asked to complete the International Physical Activity Questionnaire—Short Form (IPAQ–SF) to measure the levels of PA. Then, their weight and height were measured, and finally their spine was assessed.

### 2.4 Physical activity level measurement

Participants included in the study were physically active, as among the inclusion criteria there was the participation in any PA or sports for at least 5 years, and, in order to measure the level of PA practiced, expressed as energy expenditure (MET–minutes/week), the IPAQ–SF was administered (21). The IPAQ–SF is a standardized instrument used to assess the levels of PA practice in a population during the “last 7 days” or in the “usual week.” In detail, the questionnaire measures frequencies and durations of sitting activities, walking activities, moderate-intensity PA and vigorous-intensity PA.

Following the scoring protocol of the “Guidelines for Data Processing and Analysis of the International Physical Activity Questionnaire (IPAQ)–Short and Long Forms” and using the Compendium of Physical Activities (and subsequent updates) (22–24), participants were divided into the 3 following groups: low active (LA; <600 MET–minutes/week; n = 16); moderate active (MA; ≥600 MET–minutes/week and <3,000 MET–minutes/week; *n* = 15); high active (HA; ≥3,000 MET–minutes/week; *n* = 12).

### 2.5 Anthropometric measurements

Weight and height were recorded using a Seca electronic scale (maximum weight: 300 kg, resolution: 100 g; Seca; Hamburg, Germany) and a standard stadiometer (maximum height: 220 cm, resolution: 1 mm), respectively.

### 2.6 Spine assessment

In order to assess the spine morphology a non-invasive, 3D optoelectronic detection system using the Light Detection and Ranging (LIDAR) technology with an infrared Time-of-Flight (ToF) camera (Spine 3D; Sensor Medica, Guidonia Montecelio, Rome, Italy) was used (17–20, 25).

The validity of rasterstereography technique was previously investigated compared with X-ray and this examination has been shown to be valid for screening, monitoring scoliosis progression, follow-ups, as well as for scoliosis diagnosis (14). Furthermore, the rasterstereography technique has been shown to have excellent intra- and inter-day reliability in most parameters with the higher reliability coefficients ranging between 0.972 and 0.982 for the Intraclass Correlation Coefficient (ICC) and between 0.989 and 0.991 for the Cronbach Alpha (Cα) (26). Similarly, the rasterstereography technique has been demonstrated to have excellent intra- and inter-observer reliability of all parameters showing the maximum ICC = 0.988 and the minimum ICC = 0.918 (27). A previous study demonstrated a variability, expressed as Standard Error of Measurement (SEM), less than 1.5° and 1.5 mm for the angular and linear parameters, respectively (28).

Each participant was asked to stay 1 meter from the instrument, in upright position barefoot and with feet placed side-by-side, head in neutral position, and with the back bare-chested facing the camera of the instrument.

The anatomical landmarks were as follows: prominent vertebra (VP), right and left shoulder (SR and SL), right and left lumbar dimple (DR and DL) and the midpoint between them (DM). Based on these landmarks, the software computes specific parameters in the frontal, sagittal, and horizontal planes. The main parameters were as follows. Frontal plane—(a) Spine Length: the length of the segment from VP to DM; (b) Spine Frontal Imbalance: the length between the vertical line passing through DM and the vertical line passing through VP; (c) Spine imbalance: the angle between the line passing through VP and DM and the vertical line passing through DM; (d) Shoulder obliquity: the distance between the horizontal axis passing through SL and the horizontal axis passing through SR; (e) Shoulder tilt: the angle between the line passing through SL and SR and the horizontal axis; (f) Maximum left vertebral deviation: convexity to the left; (g) Maximum right vertebral deviation: convexity to the right; (h) Maximum left rotation surface: the angle between the line passing through the centre of the vertebral body and the apex of the spinous process and the line perpendicular to the frontal plane; (i) Maximum right rotation surface: the angle between the line passing through the centre of the vertebral body and the apex of the spinous process and the line perpendicular to the frontal plane; (j) Pelvis obliquity: the distance between the horizontal axis passing through DL and the horizontal axis passing through DR; (k) Pelvis tilt: the angle between the line passing through DL and DR and the horizontal axis; (l) Cobb curve thoraco-lumbar: the Cobb’s angle between T9 and L2; (m) Cobb curve lumbar: the Cobb’s angle between L2 and L4. Sagittal plane—(a) Spine Length: the length of the segment from VP to DM; (b) Spine Sagittal Imbalance: the length between the vertical line passing through VP and the vertical line passing through DM; (c) Spine Inclination: the angle between the line passing through VP and DM and the vertical line passing through DM; (d) Cervical Lordosis: the distance between the cervical apex and the tangent to the kyphotic apex; (e) Lumbar Lordosis: the distance between the lumbar apex and the tangent to the kyphotic apex; (f) Kyphotic Angle: the upper angle formed by the tangents to the surface at the cervico-thoracic inversion (ICT) and the thoraco-lumbar inversion (ITL) points; (g) Lordotic Angle: the upper angle formed by the tangents to the surface at ITL and the lumbosacral inversion (ILS) points. Horizontal plane—(a) Shoulder torsion: the angle of rotation of the shoulder girdle (SL-SR); (b) Pelvis torsion: the angle of rotation of the pelvis girdle (DL-DR).

For each participant, the spine assessment was conducted by the same researcher, who was an expert in the use of the instrument, during the same time slot (i.e., between 9:00 and 12:00) in order to minimize time-of-day effect.

### 2.7 Statistical analysis

Data distributions were tested using the Shapiro–Wilk’s test. Data are presented as mean ± standard deviation. Differences in spine parameters among the three groups, in the frontal and sagittal plane, were evaluated using the one-way analysis of variance (ANOVA). The Tukey’s *post-hoc* multiple comparisons test was carried out in presence of significant difference. Moreover, in case of significant difference, the eta-squared (η^2^) was carried out to measure the effect size. Mean differences in spine parameters among the three groups were calculated.

The level of significance for all statistical analyses was set at *p* < 0.05.

All statistical analyses were performed using Jamovi software; The jamovi project (2022). jamovi. (Version 2.3) [Computer Software]. Retrieved from: https://www.jamovi.org.

All figures were created using GraphPad Prism 8 (GraphPad Software Inc., San Diego, CA, USA).

A *post hoc* analysis was computed to detect the achieved sample size power, using an ANOVA design (*f* = 0.40, *α* = 0.05), using G*Power software (v. 3.1.9.2; Heinrich Heine University, Düsseldorf, Germany).

## 3 Results

The Shapiro–Wilk’s test showed that data were normally distributed.

Participants’ characteristics were as follows: LA group: *n* = 16; age: 71.38 ± 4.53 years; weight: 63.90 ± 8.98 kg; height: 1.55 ± 0.09 m; MA group: *n* = 15; age: 70.67 ± 3.74 years; weight: 66.45 ± 9.26 kg; height: 1.55 ± 0.09 m; HA group: *n* = 12; age: 69.75 ± 2.67 years; weight: 65.45 ± 8.61 kg; height: 1.56 ± 0.05 m.

The *post hoc* power analysis showed that with a total sample size of 43 participants a power of 0.61 was achieved.

Tables 1, 2 shows descriptive data of the spine parameters for each group in the frontal and sagittal plane, respectively.

**Table 1:** Descriptive data of the spine parameters in the frontal plane.

Parameter	Group	Mean ± SD
Spine length (mm)	LA	419.69 ± 34.71
	MA	413.67 ± 29.34
	HA	407.08 ± 20.48
Spine frontal imbalance (mm)	LA	−4.63 ± 9.91
	MA	−0.33 ± 9.20
	HA	−3.75 ± 10.22
Spine imbalance (°)	LA	0.66 ± 1.39
	MA	0.04 ± 1.26
	HA	0.52 ± 1.47
Shoulder obliquity (mm)	LA	−1.25 ± 12.87
	MA	2.93 ± 11.63
	HA	3.92 ± 11.52
Shoulder tilt (°)	LA	−0.20 ± 2.45
	MA	0.53 ± 2.08
	HA	0.72 ± 2.08
Maximum left vertebral deviation (mm)	LA	−4 ± 4.55
	MA	−5.67 ± 4.92
	HA	−6.17 ± 5.15
Maximum right vertebral deviation (mm)	LA	1.69 ± 2.21
	MA	1.93 ± 2.58
	HA	3.42 ± 3.68
Maximum left rotation surface (°)	LA	−11.39 ± 7.44
	MA	−12.38 ± 7.02
	HA	−13.23 ± 8.30
Maximum right rotation surface (°)	LA	2.89 ± 4.09
	MA	0.95 ± 1.90
	HA	3.28 ± 4.08
Pelvis obliquity (mm)	LA	0.06 ± 4.74
	MA	2.40 ± 4.66
	HA	2.50 ± 5.25
Pelvis tilt (°)	LA	−0.04 ± 2.60
	MA	1.33 ± 2.68
	HA	1.47 ± 3.03

LA, low active; MA, moderate active; HA, high active; mm, millimeters; °, degrees.

**Table 2:** Descriptive data of the spine parameters in the sagittal plane.

Parameter	Group	Mean ± SD
Spine length (mm)	LA	419.69 ± 34.71
	MA	413.67 ± 29.34
	HA	407.08 ± 20.48
Spine sagittal imbalance (mm)	LA	53.75 ± 17.49
	MA	36.93 ± 17.29
	HA	32.42 ± 18.31
Spine inclination (°)	LA	7.67 ± 2.76
	MA	5.05 ± 2.28
	HA	4.50 ± 2.80
Cervical lordosis (mm)	LA	42.63 ± 16.46
	MA	39.93 ± 8.83
	HA	32.67 ± 13.58
Lumbar lordosis (mm)	LA	59.38 ± 17.50
	MA	57.00 ± 12.00
	HA	51.42 ± 13.89
Kyphotic angle (°)	LA	57.04 ± 13.77
	MA	54.62 ± 11.83
	HA	51.85 ± 14.29
Lordotic angle (°)	LA	62.22 ± 34.05
	MA	46.11 ± 15.79
	HA	48.34 ± 12.15

LA, low active; MA, moderate active; HA, high active; mm, millimeters; °, degrees.

The one-way ANOVA detected no significant differences in spine parameters in the frontal plane among the 3 groups, as shown in Table 3.

**Table 3:** One way ANOVA of the spine parameters in the frontal plane among the three groups.

Parameter	*F*	df1	df2	*p*
Spine frontal imbalance (mm)	0.82	2	40	0.45
Spine imbalance (°)	0.86	2	40	0.43
Shoulder obliquity (mm)	0.76	2	40	0.48
Shoulder tilt (°)	0.71	2	40	0.50
Maximum left vertebral deviation (mm)	0.80	2	40	0.46
Maximum right vertebral deviation (mm)	1.45	2	40	0.25
Maximum left rotation Surface (°)	0.21	2	40	0.81
Maximum right rotation surface (°)	1.84	2	40	0.17
Pelvis obliquity (mm)	1.21	2	40	0.31
Pelvis tilt (°)	1.36	2	40	0.27

df, degrees of freedom; mm, millimeters; °, degrees.

In the sagittal plane, we found a significant difference on the spine sagittal imbalance parameter among the 3 groups (*F*_(2, 40)_=6.17; *p* = 0.005) (Table 4), with the highest spine sagittal imbalance in the LA group (Tables 2, 5). The effect size was η^2^ = 0.229.

**Table 4:** One way ANOVA of the spine parameters in the sagittal plane among the 3 groups.

Parameter	*F*	df1	df2	*p*	η^2^
Spine length (mm)	0.63	2	40	0.54	–
Spine sagittal imbalance (mm)	6.17	2	40	0.005	0.229
Spine inclination (°)	5.93	2	40	0.006	0.236
Cervical lordosis (mm)	1.96	2	40	0.15	–
Lumbar lordosis (mm)	1.02	2	40	0.37	–
Kyphotic angle (°)	0.52	2	40	0.60	–
Lordotic angle (°)	2.07	2	40	0.14	–

df, degrees of freedom; mm, millimeters; °, degrees.

**Table 5:** Tukey’s *post-hoc* multiple comparisons test for the spine sagittal imbalance (mm).

Multiple comparisons	Mean difference	*p*
LA vs. MA	16.82	0.030
LA vs. HA	21.33	0.008
MA vs. HA	4.52	0.787

LA, low active; MA, moderate active; HA, high active.

**Figure 1:**
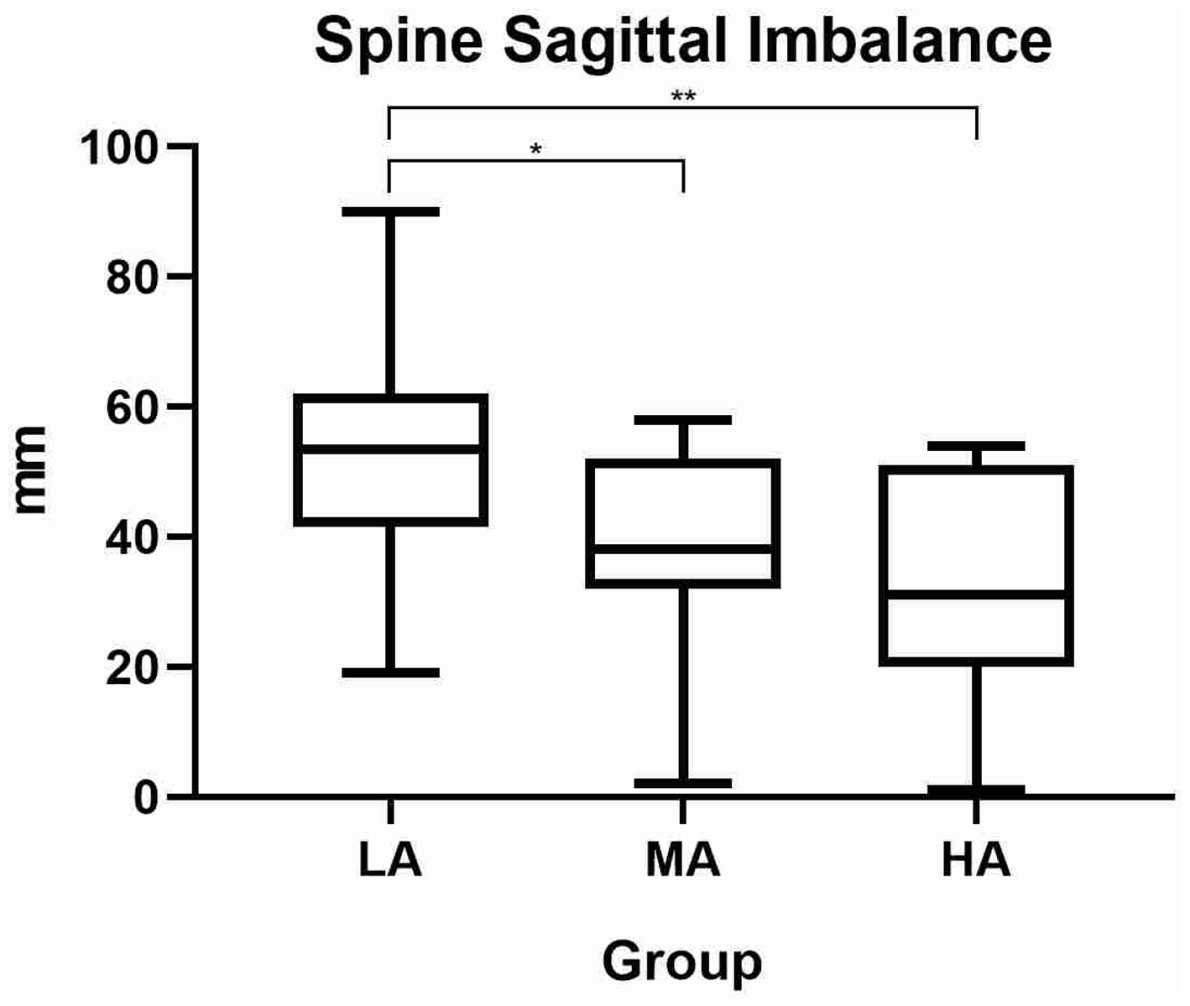
Spine sagittal imbalance differences among the three groups. LA, low active; MA, moderate active; HA, high active; mm, millimeters.

The Tukey’s *post-hoc* multiple comparisons test showed a significant difference between LA and MA group (*p* = 0.030), and between LA and HA group (*p* = 0.008), as reported in Table 5 and Figure 1.

Furthermore, in the sagittal plane, we detected a significant difference in the spine inclination parameter among the 3 groups (*F*_(2, 40)_=5.93; *p* = 0.006) (Table 4), with the highest spine inclination in the LA group (Tables 2, 6). The effect size was η^2^ = 0.236.

**Table 6:** Tukey’s *post-hoc* multiple comparisons test for the spine inclination (°).

Multiple comparisons	Mean difference	*p*
LA vs. MA	2.62	0.021
LA vs. HA	3.16	0.008
MA vs. HA	0.54	0.854

LA, low active; MA, moderate active; HA, high active.

**Figure 2:**
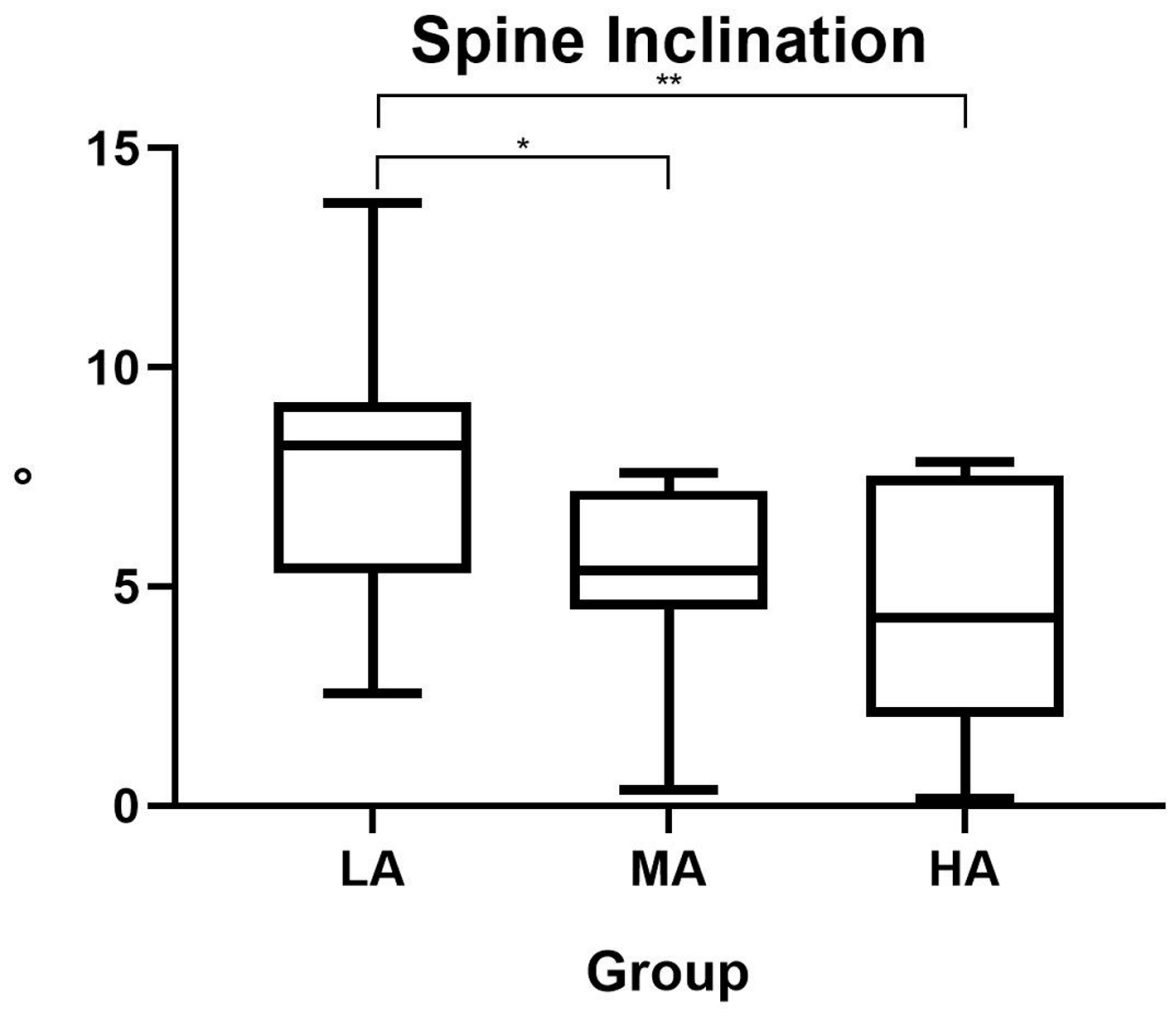
Spine inclination differences among the three groups. LA, low active; MA, moderate active; HA, high active; °, degrees.

The Tukey’s *post-hoc* multiple comparisons test showed a significant difference between LA and MA group (*p* = 0.021), and between LA and HA group (p = 0.008), as reported in Table 6 and Figure 2.

## 4 Discussion

The aim of this study was to analyze any spine differences in older women comparing subjects with different levels of PA. Our results showed that older women who engaged in lower levels of PA exhibited some altered spinal sagittal parameters compared to peers with moderate and high levels of PA. Specifically, we found an increasing sagittal imbalance of the spine as the level of PA decreased, that is, from high active to low active. Similarly, we detected the same trend for the sagittal inclination of the spine with the highest angle in the low active group. Moreover, the differences found are clinically meaningful. In fact, the significant mean differences we detected exceed the range of SEM of the rasterstereography technique (28). These alterations reflect a forward displacement of the trunk and consequent less efficient body posture (29, 30).

Physiological and biomechanical changes during aging such as a lumbar lordosis reduction and a thoracic kyphosis increase, leading to pelvic retroversion, have been documented (31). The existing literature has documented the importance of spine assessment in the sagittal plane, as spinal sagittal malalignment may reflect a loss of the physiological curves of the spine, with possible forward tilt of the trunk, and possible consequent posterior pelvic rotation (6, 10, 32). Indeed, the assessment of the spine in the sagittal plane can be useful in order to have an image of the spine shape, and to monitor differences in spine alignment and compensation (6). As a matter of fact, spinal balance can depend on muscle response of the trunk to maintain stable upright posture (33). In fact, a research group demonstrated that a sagittal vertical axis higher than 40 mm indicates a poor sagittal balance and higher values of this parameter indicate poorer sagittal alignment (34, 35). A sagittal vertical axis higher than 40 mm significantly increases the number of falls (36). In the sample we recruited, the LA group showed a spine sagittal imbalance of 53.75 ± 17.49 mm and this can represent a significant clinical risk (37). As a matter of fact, spinal sagittal alignment is a key factor for preventing and managing spinal disorders, especially during aging (29). Our results also showed that the LA group had a spine inclination of 7.67 ± 2.76 ° resulting in significant health risks that negatively impact the quality of life (30). In fact, an optimal spinal sagittal balance is fundamental for maintaining a neutral standing posture during daily activities (29).

These results could find practical application in evaluating spine differences based on age, gender, or PA practice. In fact, previous research showed age- and gender-related differences in spinal sagittal morphology. For example, a study by Yukawa et al. (38) showed changes in sagittal alignment according to gender and age in a large sample of asymptomatic individuals. In particular, in line with our results, the authors found, among their findings, an increase in the sagittal vertical axis as physiological change with advancing age (38). In the same way, Imagama et al. (39) reported that physical functions are negative correlated with both age and spinal inclination angle in middle-aged and elderly subjects and, among other, these could affect the quality of life. Moreover, the participants who were engaged in physical exercise exhibited higher levels of physical characteristics including greater thoracic spinal range of motion (ROM) and back muscle strength as well as good spinal balance underlining the importance of physical exercise on the morpho-functional characteristics of the spine (39). Indeed, the practice of leisure time PA, regular PA, structured exercise, and sports, can protect against spine degeneration, can prevent from sarcopenia and osteopenia in postmenopausal women (40–42). It is widely known that regular PA appears to protect against spinal deterioration by contributing to the maintenance of muscle mass and strength, joint flexibility, and posture (43, 44). In fact, the benefits of PA on the musculoskeletal health of the back are well documented in the literature (45, 46). It seems that the compressive strength of the spine tends to increase with the level of PA practiced and that PA strengthens both the vertebrae and the discs (47). An interesting study by Borg-Stein et al. (48) explored the aging spine in sports showing that the benefits of regular sports engagement overcome the potential risks of spine degeneration in middle-aged athletes. A seminal study in this field showed that in a sample of women aged 55 to 75 who had started physical exercise regularly at age 50, bone mineral density was significantly higher than that of women of an age-matched sample who had not engaged in physical exercise (48, 49).

Most of the participants recruited for this study had been participating in walking groups, and others in postural gymnastics or Pilates. Indeed, different types of exercise are effective for spine health as aerobic exercises, e.g., walking or swimming, thanks to the fact that these activities offer low impact on the spine (50, 51), in contrast to activities that can have high impact on the spine such as running (52). For example, a recent systematic review demonstrated that, due to compression pushing water content out of the disc, running has a negative impact on intervertebral discs (52). Furthermore, compression of the intervertebral discs can increase as the intensity of running increases (53). While, the types of load useful for intervertebral discs are dynamic, axial, at slow to moderate movement speeds, such as in walking (54). As for swimming, it has been demonstrated that can improve bone mineral density in postmenopausal women, especially in long-term, and this can prevent spinal deformities (51, 55). As a matter of fact, low bone mineral density is a predictor of spinal deformities (55–57).

Among the types of exercise recommended, the effectiveness of strength exercises for preventing and managing spine malalignment or spine disorders, e.g., Pilates or Yoga, is well demonstrated by several research groups (44, 58–61). For example, Pilates resulted an effective method for reducing pain and improving flexibility, as well as static and dynamic endurance in subjects with lumbar disc herniation (59). Similarly, the stretch and strength-based Yoga exercise showed a significant pain reduction in subjects with lumbar disc herniation (60). Based on previous studies, the improvement in back muscle strength as well as in core muscle strength through exercise can prevent spinal degeneration and prevent spinal sagittal malalignment (36, 62, 63). As reported in a recent umbrella systematic review, combined resistance exercises are effective in preserving bone mineral density of the lumbar spine, and the concurrent training showed significant improvements (64). Indeed, the literature agrees that combining various exercise programs has a positive effect on lumbar spine bone mineral density (65).

In contrast, it is well recognized that static sitting posture increases the compression of the lumbar intervertebral discs (66). These studies demonstrate that the health and the morphology of the spine can depend on the type and level of PA practice, supporting our research hypothesis.

### 4.1 Strengths and limitations

The originality of this study was to consider the level of PA practice as an influencing factor for spinal sagittal alignment, underlying that the amount of PA may play a key role in preventing spinal sagittal imbalance.

Among the limitations it must be mentioned that it is a cross-sectional study limiting causal inference. Furthermore, it must be mentioned that the sample size does not allow to generalize the results. In fact, the *post hoc* analysis showed that the sample size achieved a power of 0.61.

### 4.2 Practical implications

This study emphasizes the effectiveness of PA on the aging of the spine. Moreover, it should be underlined that the use of non-invasive techniques to screen and monitor spine morphology can be useful to avoid radiographic radiation when radiographic examination is not justified, and to adopt the rasterstereography technique in routine clinical use.

## 5 Conclusion

Our findings that low active women showed higher values of spine sagittal imbalance and spine inclination compared to women engaged in moderate or high levels suggest that PA may contribute to maintain spinal sagittal alignment and preserve spinal sagittal balance. This could probably be explained by the fact that PA leads to the strengthening of the back muscles, including stabilizing muscles of the spine, which could slow down the physiological progression of sagittal imbalance that occurs with advancing age. Although PA can prevent the aging of the spine, further studies should clarify to what extent PA can affect spinal alignment.

## Data Availability

The raw data supporting the conclusions of this article will be made available by the authors, without undue reservation.
